# New methods, old questions: advancing the study of unconscious perception

**DOI:** 10.3389/fpsyg.2025.1626223

**Published:** 2025-08-20

**Authors:** Mikel Jimenez, Antonio Prieto, Pedro R. Montoro, José Antonio Hinojosa, Markus Kiefer

**Affiliations:** ^1^Departamento de Psicología Básica I, UNED, Madrid, Spain; ^2^Instituto Pluridisciplinar, Universidad Complutense de Madrid, Madrid, Spain; ^3^Departamento de Psicología Experimental, Procesos Psicológicos y Logopedia, Universidad Complutense de Madrid, Madrid, Spain; ^4^Centro de Investigación Nebrija en Cognición (CINC), Universidad de Nebrija, Madrid, Spain; ^5^Department of Psychiatry, Ulm University, Ulm, Germany

**Keywords:** awareness, consciousness, unconscious perception, visual masking, awareness thresholds

## Abstract

Since the early experimental studies of the late 19th century, research on unconscious perception has been shaped by persistent methodological challenges and evolving experimental approaches aimed at demonstrating perception without awareness. In this review, we will discuss some of the most relevant challenges researchers have faced in demonstrating unconscious perception, and examine how different measures of awareness (e.g., objective vs. subjective) yield different awareness thresholds—often leading to two alternative approaches to demonstrating unconscious perception. We will further explore new methodologies in the field, such as regression-based Bayesian modeling, sensitivity vs. awareness (SvA) curves derived from General Recognition Theory (GRT), the liminal-prime paradigm, and two-interval forced choice (2IFC) designs. Finally, we emphasize the need for brain-based approaches to unconscious perception and discussed some promising studies in this area, while also highlighting the role of individual differences and alternative frameworks such as predictive coding and active inference views in future research. Overall, the new approaches and methodologies discussed here will advance the field by addressing the challenges inherent in demonstrating cognition in the absence of awareness.

## 1 Unconscious perception: a journey of methodological struggles

To what extent can information outside our consciousness influence our behavior? Since the early experimental studies in the late 19th century (Pierce and Jastrow, 1884; Sidis, 1898), research on unconscious perception has been shaped by persistent methodological challenges and shifts in experimental approaches to demonstrate perception without awareness^1^ (Jimenez et al., 2023; Rothkirch and Hesselmann, 2017; Shanks and John, 1994; Yaron et al., 2024). As Michel (2023) illustrates, the scientific study of the unconscious mind seems to parallel the myth of Sisyphus: Researchers advance the ‘unconscious rock’ toward the summit, only to see it regress, requiring the effort to begin anew. In this review, we focus on the field of unconscious (visual) perception. Note, however, that the study of unconscious cognition expands to other domains, such as language, emotion, attention or memory (see Mudrik and Deouell, 2022, for a review). Within memory research, for example, the processing of implicit memories ab Cheesman out behavior or even the activation of memories without their expression (an aspect equivalent to how encoded information affects behavior without conscious access) has been demonstrated (Delorenzi et al., 2014). A substantial body of research has examined how implicit information—particularly emotional stimuli—can affect decision-making processes without entering conscious awareness (Bernal et al., 2022; Morris et al., 1998). The field of implicit learning is also a fruitful area of research within experimental psychology (see Shanks, 2005, for a review).

This struggle is evident in the shifting methodologies over time. Early studies, for instance, relied on introspective reports as a valid scientific approach to studying mental states. Under this approach, an introspective report of ‘no awareness’ combined with above-chance performance on an objective (e.g., stimulus discrimination) task was taken as evidence for unconscious perception (the so-called blindsight phenomenon). Throughout the first half of the 20th century, researchers in both visual and auditory domains contrasted subjective and objective tasks to dissociate awareness and performance (Baker, 1938; Miller, 1939; Stevens, 1935; Stevens et al., 1937; Williams, 1938; see Adams, 1957, for a review).

The approach shifted following Eriksen’s (1960) methodological critique. He strongly rejected introspection as a valid measure of awareness, arguing that subjective reports might primarily reflect a participant’s response criterion rather than subjective experience, while being highly susceptibility to response biases (Eriksen, 1960; Jimenez et al., 2020; Kouider and Dehaene, 2007). Eriksen’s critique highlighted the importance of valid measures of (un)awareness. This led to the development of the classic dissociation paradigm (Reingold and Merikle, 1988; Schmidt and Biafora, 2024), which aimed to demonstrate unconscious perception by comparing performance, typically in terms of priming or facilitation effects (indirect measure), and an awareness measure on the same stimuli (direct measures). Yet this new approach also raised several methodological concerns (Holender, 1986; Kouider and Dehaene, 2007; Jimenez et al., 2023). Perhaps one of the most relevant raised doubts about whether a subset of ‘conscious’ participants was influencing the observed perfomance effect. (Harris et al., 2011; Jimenez et al., 2017; Muscarella et al., 2013; Shanks, 2017). Furthermore, this procedure rests on the acceptance of the null hypotheses with regard to zero awareness, which is problematic for statistical reasons (Schmidt, 2007).

Different solutions to the dissociation paradigm have been proposed over the years (e.g., Snodgrass et al., 2004; Reingold, 2004). One solution is to look for double dissociations in terms of qualitatively different effects of an experimental manipulation on direct and indirect measures, for instance a manipulation produces an increase of the direct measures, but a decrease of the indirect measure (Schmidt, 2007). In addition, the development of different new experimental techniques, such as regression-based methods (Greenwald et al., 1995; Goldstein et al., 2022) or Jacoby’s process dissociation procedure (PDP) (Jacoby, 1991) tried to overcome some of these problems.^2^ However, proving the existence of perception without awareness has proved more challenging than anticipated. In the following sections, we will discuss the main obstacles researchers have faced to prove perception in the absence of awareness, as we present some interesting new methodological approaches that may advance research within the field.

## 2 Different measures of awareness produce different thresholds of awareness

Evaluating unconscious processing requires a reliable measure of conscious perception. Yet the two most used methods−subjective reports and objective forced-choice stimulus detection/discrimination−do not always agree, as observers can sometimes discriminate stimuli they rated invisible. It is a long-held view that subjective and objective measures of awareness seem to reflect different thresholds of awareness (for a critical discussion, see Kiefer and Kammer, 2024).

*Awareness thresholds* refer to the minimum stimulus conditions required for conscious perception (Merikle et al., 2001). When using a subjective measure of visual awareness −such as asking participants to report their visual experience− a subjective threshold is established, whereby a participant is considered unaware if they report having ‘no perception’ of the stimulus (Timmermans and Cleeremans, 2015). In contrast, objective measures define unawareness based on the inability to discriminate between stimuli (e.g., chance performance) in an objective task. The distinction between objective and subjective thresholds has commonly led to the differentiation of different stages in visual processing: a participant may be *objectively unconscious* of a stimulus when their discrimination performance is at chance level, *subjectively unconscious* if they report ‘no perception’ of a stimulus, with either chance or above-chance discrimination performance, and (partially or fully) *conscious*, which would imply both above-chance discrimination and subjective ‘seen’ reports (e.g., both above objective and subjective thresholds; Lamme, 2020).

Under this classical approach, the *objective threshold is* often considered a more conservative estimate of awareness (Merikle et al., 2001) and consciousness development is considered as linear, from objectively unconscious to subjectively unconscious and ultimately conscious states. This long-held view, however, may be oversimplified and inconsistent with recent findings. For example, Kiefer et al. (2023a, 2023b) found that the thresholds for objective and subjective measures of awareness converge, a pattern also noted by others (Jimenez et al., 2023; Schmidt and Biafora, 2024). Furthermore, Kiefer and Kammer (2024) argue that the relationship between subjective and objective measures is complex, with subjective thresholds sometimes preceding objective ones. These authors propose three possible scenarios for the relationship between subjective and objective awareness thresholds. In the first scenario, subjective thresholds lag behind objective ones. Here, early objective performance might reflect fast unconscious processing, while subjective measures require more time for visual consolidation and a specific conscious experience. Alternatively, only subjective measures could depend on meta-cognitive evaluations, adding an extra processing step. In the second scenario, objective measures lag behind subjective ones, as subjective measures capture a broader range of sensory experiences. In the third scenario, subjective and objective thresholds converge (see Kiefer and Kammer, 2024, for a discussion). In fact, there is empirical support for all three scenarios. Among these lines, Prieto et al. (2025) recently introduced a novel paradigm where both objective and subjective awareness of the prime was gathered on a trial-by-trial basis. Their results showed that, when converted to a common sensitivity measure (*d’*), objective and subjective awareness measures collected during both the priming and visibility blocks showed comparable values within the same block, strong correlations both within and across blocks, and, crucially, were responsive to variations in prime visibility linked to attentional differences arising from distinct task demands (Prieto et al., 2025). As discussed, this new evidence suggests that when converted to a common sensitivity measure (*d’*), objective and subjective awareness measures might provide a comparable index of observers’ awareness.

In sum, the observed variability might arise from methodological differences across studies, where objective measures may be influenced by unconscious response tendencies depending on the task. Yet for the research of unconscious perception to advance, the specific relation between subjective and objective thresholds of awareness needs to be fully understood.

## 3 Testing unconscious perception: two alternative approaches

The debate on demonstrating perception without awareness seems to rest largely on differing assumptions about how to establish evidence for unconscious perception. Using an *objective threshold* to measure awareness assumes that any *d’* > 0 (i.e., above-chance stimulus discrimination) indicates conscious awareness of the stimuli. In contrast, a *subjective threshold*—based on participants’ self-reported lack of awareness—interprets any *d’* > 0 as evidence of unconscious perception, provided it coincides with a “no awareness” report.

As discussed above, early attempts in the field leaned on the implementation of subjective thresholds to demonstrate subliminal perception (Pierce and Jastrow, 1884; Sidis, 1898). A classic example is the work by Pierce and Jastrow (1884), who had participants compare finger pressures and rate their subjective experience on a 0 to 3 scale. Despite reporting no awareness, participants performed above chance in distinguishing the stimuli, suggesting non-conscious influences on behavior. Similarly, Sidis (1898) found that participants who reported seeing only a faint blur of alphanumeric characters could still identify them better than chance, reinforcing the idea of unconscious perception. This approach quickly became popular and several studies on the visual and auditory domains followed during the first half of the 20th century (Baker, 1938; Miller, 1939; Stevens, 1935; Stevens et al., 1937; Williams, 1938; see Adams, 1957, for a review). A longstanding concern has been that the subjective measures used by Peirce, Jastrow, and later researchers to assess awareness are invalid (Eriksen, 1960). While some researchers (e.g., Marcel, 1983) later claimed to employ an objective measure of awareness to demonstrate unconscious cognition (e.g., semantic priming), this turned out not to be the case. In addition, this study considered performance below 60% accuracy as indicative of unawareness, despite chance level being 50%. When awareness was assessed using stricter criteria, Cheesman and Merikle (1985) failed to replicate Marcel’s findings (see also Kouider and Dehaene, 2007). Accordingly, some views challenge the existence of unconscious perception (Persuh, 2018; Phillips, 2021), proposing that a conscious-perception-only model should be treated as the default or null hypothesis.

After Eriksen (1960), participants’ introspection was no longer accepted as a valid measure of awareness. This led to the development and adoption of a more rigorous framework, which would operationalize the absence of awareness through an objective awareness threshold (Reingold and Merikle, 1988; Schmidt and Biafora, 2024). Under this new approach, any above-chance stimulus discrimination (i.e., *d’* > 0) would indicate conscious awareness of the stimuli, and was mainly implemented through a dissociation paradigm, where observers completed a discrimination task on a probe stimulus (performance measure) first and subsequently assessed their awareness of the primes (awareness measure). If prime discrimination is at chance but those same invisible primes facilitate (or interfere with) probe responses (e.g., priming effects are found), it suggests the visual system processes information without conscious awareness. Several problems with this new framework were quickly brought forward. According to the *retrospective assessment* or *immediacy problem*, the awareness measures may not capture information processed unconsciously due to memory decay (Shanks and John, 1994; Newell and Shanks, 2014). The *information relevance criterion* stresses that awareness measures must assess information directly related to task performance; and the *sensitivity criterion* requires performance and awareness measures to be equally responsive to relevant stimuli. One of the main issues is that group visibility is often not at strict chance level, which has led to *post-hoc data selection*, where only participants below a certain awareness threshold are analyzed (Rothkirch et al., 2022; Shanks, 2017). This produces statistical artefacts such as regression to the mean (RttM).^3^ Lastly, the absence of significant awareness effects was often misinterpreted as evidence of unconscious processing, because it rests on the problematic acceptance of the statistical null hypothesis as outlined above (Reingold and Merikle, 1988; Schmidt and Vorberg, 2006; Shanks, 2017; see Jimenez et al., 2025, for a review). Beyond these methodological issues, a deeper concern lies in the assumption underlying the use of objective thresholds of awareness: while any d′ > 0 (i.e., above-chance stimulus discrimination) is typically interpreted as evidence of conscious awareness of the stimuli, it cannot be ruled out that such above-chance performance may instead reflect the very unconscious influences it aims to demonstrate, rather than conscious perception per se (Merikle et al., 2001). As clearly put by Micher et al. (2024), the key debate lies on whether the discrepancy between objective and subjective thresholds reflects a contamination of subjective reports by conscious perception or whether forced-choice discrimination performance is influenced by unconscious processing. It’s important to recognize that questioning whether forced-choice discrimination is affected by unconscious processing presupposes the existence of unconscious processing (for a discussion, see Koivisto and Neuvonen, 2020). As discussed above, modern psychology has gone through recurring cycles of excitement about unconscious perception, followed by waves of skepticism and criticism (Holender, 1986; Overgaard and Timmermans, 2010; Irvine, 2012; Michel, 2019). Some views challenge the existence of unconscious perception (Persuh, 2018; Phillips, 2021), proposing that a conscious-perception-only model should be treated as the default or null hypothesis. According to this, it is reasonable to begin with skepticism toward claims of unconscious perception, and it is therefore up to those who support the existence of unconscious perception to present strong and convincing evidence to support their position (Phillips, 2021).

## 4 Breaking the stalemate: new methodological approaches to subliminal perception

To overcome some of the issues with the dissociation paradigm, several studies implemented subjective awareness reports within the ‘performance measure’ itself (e.g., Kiefer et al., 2023a; Sabary et al., 2020; Van den Bussche et al., 2013). This allows for participants’ awareness reports being gathered on a trial-by-trial basis within the priming task, addressing the *immediacy problem* by collecting both performance and awareness measures simultaneously. Subjective measures, unlike objective ones, are more directly tied to conscious perception (Dehaene and Naccache, 2001; Merikle et al., 2001; Persuh, 2018). However, using subjective measures in masked priming has methodological drawbacks, including susceptibility to response biases (Eriksen, 1960; Merikle et al., 2001) and criterion confounds (Bachmann, 2015; Kahneman, 1968). Shifts in response criteria can bias awareness reports, even within the same experiment (Jimenez et al., 2020, 2023). Moreover, incorporating online subjective reports introduces a dual-task paradigm, potentially slowing probe latencies (Jimenez et al., 2023; Kiefer et al., 2023a), weakening prime-probe links (e.g., Ansorge et al., 2011) and even abolishing priming effects (Kiefer et al., 2023a; Wentura et al., 2025). This can also induce a conservative awareness criterion (‘c’ in SDT; Macmillan and Creelman, 2004), likely due to the high cognitive load and difficulty in attending to both prime and probe stimuli.

Overall, the methodological challenges highlighted above stress the ongoing need for better approaches to uncover unconscious stimulus processing. Several new techniques have recently emerged, some refining the classic dissociation paradigm (Goldstein et al., 2022), particularly requesting double dissociations (Schmidt, 2007), and others implementing new methodologies to assess visual stimulus discrimination without awareness based on participants’ self-reported lack of awareness (e.g., subjective thresholds; Pournaghdali et al., 2023). For example, Goldstein et al. (2022) proposed a Bayesian generative model to estimate the intercept in a regression model while accounting for error in awareness and effect measurements. This model builds on Greenwald et al.'s (1995) regression method, which addresses a common pitfall within the field (e.g., post-hoc data selection; Yaron et al., 2024) and helps model unconscious priming effects by adjusting prime awareness values. However, previous studies have shown that the Greenwald regression often overestimates the unconscious effect (Dosher, 1998; Klauer et al., 1998). To resolve this, Goldstein et al.’s Bayesian model corrects for measurement errors in both awareness and effect measurements, offering a more accurate estimate of the unconscious priming effect (Jimenez et al., 2023; Prieto et al., 2025).

A novel method by Pournaghdali et al. (2023) reinterprets sensitivity measures (*d’*) to reflect performance in the prime visibility task rather than degree of awareness. In this work, participants discriminated emotional expressions in masked faces, with online subjective awareness measures collected. Using General Recognition Theory (GRT), the authors created sensitivity vs. awareness (SvA) curves, which linked sensitivity (*d’*) for emotional expression discrimination to the relative likelihood of no awareness (RLNA). A *d’* > 0 with high RLNA indicated unconscious processing, leading Pournaghdali et al. (2023) to conclude that unconscious processing of facial emotion occurred, evidenced by sensitivity above chance levels in areas with low awareness probability.

Micher et al. (2024) revisited a dissociation in masked response priming from the liminal-prime paradigm (Avneon and Lamy, 2018; Kimchi et al., 2018; Lamy et al., 2019; Micher and Lamy, 2023), where invisible primes affect only fast responses (50% of fastest trials; RTs ≈ 550 ms), while visible primes influence both fast and slow responses (50% of slowest trials; RTs ≈ 700 ms). Their results replicated this pattern: consciously perceived primes produced priming across all response times, whereas “invisible” primes did so only in fast trials. Notably, discrimination of “invisible” primes was above chance, suggesting that forced-choice responses may reflect unconscious processing rather than true awareness.

Note that the different approaches presented to this point may still face persistent methodological limitations. Goldstein et al. (2022) acknowledge that their framework, while modeling measurement error, might still misattribute effects to non-conscious processing if a few conscious trials—especially in the so-called “subliminal” phase—influence the results. In their simulations, they argue it is unlikely but concede it’s possible. In addition, Goldstein’s method hinges on the accuracy of the awareness task. If this task is too insensitive (e.g., due to being overly difficult or relying on poor constructs), participants may appear unaware when they actually have some awareness. That can mislead the framework into labelling effects as unconscious.

Pournaghdali et al.'s (2023) Sensitivity vs. Awareness (SvA) framework also faces some limitations. In Experiment 1, the authors themselves note that the low number of “aware” trials may constrain the robustness of their GRT model fit and subsequent SvA curve estimation. When key regions in the awareness continuum are under-sampled, the model’s inferences—especially about nonconscious sensitivity—may be unstable or overfit. In addition, this approach involves fitting multiple GRT variants and selecting the best via AIC, but with high fit percentages (~98–99%) and many parameters, there’s a risk the model is capturing noise or idiosyncrasies rather than genuine underlying processes. Overfitting could lead to spurious sensitivity in low-awareness regions. In addition, the SvA curve relies on the “optimal observer” criterion to demarcate low vs. high likelihood of awareness, a boundary that depends on assumptions about noise distributions and observer optimality. If those assumptions are incorrect—as could happen across individuals or conditions—the demarcation becomes somewhat arbitrary, affecting what is considered “unconscious” processing.

Results by Micher et al. (2024) could be alternatively interpreted, as the primes rated as “invisible” could have been processed in manner that generated a weaker signal (due to variations in the level of attention from trial to trial, for example), showing effects only on fast trials. In addition, the content of consciousness may have differed in those trials, resulting in “invisible” categorization—a possibility that relates back to criterion confounds associated with subjective measures of awareness.

The confidence forced-choice paradigm or two-interval forced choice (2IFC) paradigm has been recently proposed as a bias-resistant method for studying confidence, where participants make two perceptual judgments and select the one they believe is more accurate (Barthelmé and Mamassian, 2010; Mamassian, 2016, 2020; Peters and Lau, 2015; de Gardelle and Mamassian, 2014). Using this novel approach, Peters and Lau (2015) found no indication of unconscious processing, as indexed by above-chance accurate responding for stimuli rated as subjectively unaware; whenever subjects discriminated the stimuli above chance, they were subjectively aware of the stimuli. Note that some questions have been raised about this method (see Phillips, 2021, for a discussion). While considered largely free from response biases (Mamassian, 2020; Michel, 2023; see Jimenez et al., 2025, for a review), it has been criticized for the “criterion content fallacy”—mistakenly assuming that conscious awareness of any stimulus aspect implies awareness of task-relevant features (Michel, 2023; Rajananda et al., 2020). This issue extends to retrospective awareness reports like the perceptual awareness scale (PAS), where minimal visibility ratings (e.g., “brief glimpse”) are often treated as conscious perception, even though unconscious processing of task-relevant features remains possible (Bergström and Eriksson, 2014; King et al., 2016; Soto et al., 2011; Trübutschek et al., 2019). To address this issue, an enhanced version of the 2IFC paradigm has been developed (Amerio et al., 2023; Elosegi et al., 2024; Rajananda et al., 2020), in which both intervals contain stimuli but only one includes the task-relevant feature. If participants discriminate above chance despite reporting equal visibility across intervals, this is taken as evidence of unconscious perception (Amerio et al., 2023).

## 5 Neural correlates of unconscious perception: the need for brain-based evidence

A comprehensive understanding of perception without awareness should take advantage of recent developments in neuroimaging techniques. Ideally, we could aim to identify the neural correlates associated to different unconscious states: the neural correlates for fully unconscious stimuli, those associated with objectively unconscious stimuli, and the neural correlates for subjectively unconscious stimuli. Using functional magnetic resonance imaging (fMRI), Stein et al. (2021) have linked subjective and objective awareness states to distinct neural patterns. The findings suggest that while both objectively and subjectively invisible stimuli are represented in the visual cortex, the extent of unconscious processing depends on the measurement method. For subjectively invisible stimuli, a posterior-to-anterior gradient is observed in the visual cortex, with stronger category information in the ventrotemporal cortex than in early visual cortex. In contrast, for objectively invisible stimuli, category information remains nearly the same from early visual areas to object- and category-selective regions. However, as mask contrast was much higher for the objectively invisible than for the subjectively invisible condition, the interpretation of these results in terms of a dissociation of the neural substrate of subjective vs. objective invisibility is compromised. While more research is needed on brain activity linked to unconscious states, these findings may refine current neural theories of consciousness.

The use of machine learning (ML) models in neuroimaging analysis has opened new avenues for studying consciousness (Haynes, 2009). These models are not only advancing research on conscious experience but are also being applied to unconscious processing. A recent proposal suggests that brain-based measures may be used to pinpoint the presence of unconscious knowledge associated with null perceptual sensitivity (Soto et al., 2019). This method isolates unconscious representations by analyzing brain activity patterns when behavioral measures show no sensitivity. Techniques include transfer learning—where models trained on conscious items identify unconscious knowledge—and computational comparisons of conscious and unconscious representations. Supporting this, Mei et al. (2022) decoded unconscious image categories (living vs. non-living) from distributed multi-voxel patterns in the ventral visual pathway and parieto-frontal regions.

A complementary research line investigates dissociations between subjective and objective measures of visual awareness based on their neural foundations (Di Gregorio et al., 2022; Di Luzio et al., 2022; Trajkovic et al., 2023; Mazor et al., 2022). Using decoding analyses on functional brain imaging data, Mazor et al. (2022) found that prefrontal representations of subjective visibility are influenced by decision confidence, emphasizing the need to control for confidence in awareness studies. Novel methods integrating electrophysiology (EEG) and rhythmic transcranial magnetic stimulation (TMS) have further revealed a double dissociation in alpha activity, linking frequency to spatiotemporal sampling and amplitude to subjective sensory interpretation (Di Gregorio et al., 2022). These advances underscore the potential of brain-based approaches in shaping future research on consciousness.

## 6 The role of individual differences in consciousness research

A largely overlooked topic in unconscious perception research concerns the extent to which unconscious effects vary across individuals. Although variability in unconscious effects is frequently observed, it is not always systematically reported in the literature (Yaron et al., 2025). A notable exception comes from studies that use regression methods to estimate unconscious priming effects (see Greenwald et al., 1995; Goldstein et al., 2022, for reviews of regression-based approaches), which often provide individual-level data. Inspection of regression plots from these studies shows substantial variability in priming effects among participants with near-zero awareness scores. Some individuals display strong priming in the expected direction, while others show no effect—or even effects in the opposite direction (Draine and Greenwald, 1998; Greenwald et al., 1995, 1996; Klauer et al., 2007).^4^ Recently, genetic dispositions such as the BDNF Val66Met polymorphism and personality traits have been found to be related to the magnitude of unconscious semantic priming effects (Sanwald et al., 2020).

This variability may stem from at least two sources: (1) low reliability and high measurement error in awareness estimates (Vadillo et al., 2022), and (2) genuine individual differences in unconscious or subliminal processing. Even studies that apply improved measurement and correction methods continue to report high intersubject variability among participants deemed unaware (Jimenez et al., 2023; Prieto et al., 2025; Sanwald et al., 2020). Thus, while measurement error contributes to the noise, true individual differences in unconscious processing likely play a significant role and should not be ignored. It is worth noting that some differences may also arise from subjects’ memories, knowledge, heuristics, and/or beliefs (Gigerenzer and Goldstein, 2011; Parasuraman and Jiang, 2012). Beyond the physiological or genetic differences that may explain differences in processing in general, cognitive processes may vary interindividually as a consequence of the functionality of the cognitive system such as working memory capacity, leading to interindividual differences in the magnitude of cognitive processing (Kiefer et al., 2005; Megías et al., 2021).

Some research has explored the neural correlates of these individual differences, including electrophysiological activity, brain oscillations, neurotransmitter levels, and grey matter density (Boy et al., 2010; Cohen et al., 2009; Martens et al., 2006; van Gaal et al., 2011). A key consequence of such variability is the difficulty in interpreting null results: it becomes unclear whether they reflect a genuine lack of effect at the individual level or the averaging of opposing effects across participants.

To address this ambiguity, researchers have begun using statistical methods that are robust to variability in effect direction. For example, Yaron et al. (2025) used 26 prior datasets and applied several non-directional statistical approaches that avoid assumptions about effect consistency across individuals: (1) the prevalence global null test, (2) the qualitative individual differences approach, and (3) the omnibus ANOVA. They also introduced two new non-directional, non-parametric methods: the Sign Consistency test and the Absolute Effect Size test. While only 3 of the 26 datasets showed no detectable effects, their findings highlight the potential of these methods for more nuanced interpretations of null results in unconscious processing research.

## 7 Predictive coding and the active inference framework as a model for (un)consciousness

Although its roots trace back to Helmholtz’s idea of unconscious inference, *predictive coding* is a relatively recent framework based on a Bayesian account of brain function. The theory posits that the brain acts as a hypothesis-testing machine, continuously working to minimize the error between its predictions and the sensory input it receives from the environment (Hohwy, 2013; Parr et al., 2019). To achieve this, the brain uses a hierarchical internal generative model of the environment (i.e., hidden causes) to predict incoming sensory data and to update this model when its predictions fail (Friston and Kiebel, 2009). Within this view, perception is conceived as an active process of hypothesis testing (*active inference*), in which actions function as “experiments” that gather evidence to resolve uncertainty among competing models of the world (Friston et al., 2015). The brain’s primary goal is to infer the most likely causes of sensory signals—the Bayesian *posterior*—based on prior beliefs (*priors*) and sensory evidence (*likelihood*) (Mudrik et al., 2025).

Although predictive coding and the Active Inference Framework were not originally designed as theories of consciousness, they offer a promising foundation for making testable predictions about aspects of (un)conscious processing (Hohwy and Seth, 2020). The central idea emerging from this research is that perceptual contents are generated by the brain’s model of the causes of its sensory inputs, and that the phenomenological quality of experience is determined by the nature of the predictions involved. Changes in conscious experience thus reflect changes in the inferred state of the body, brain, or external world (Hohwy and Seth, 2020; Mudrik et al., 2025). According to this view, conscious mental states are associated predictions about the causes of sensory input, and the *contents* of consciousness correspond to the outcome of these predictions—not the inferential process itself, which is unconscious by nature (Seth and Bayne, 2022). In contrast, unconscious contents are those in which the inferences do not reach the level of precision or integration necessary for conscious access.^5^

Predictive coding and active inference hold significant potential to unify experimental findings across conscious and unconscious perception, thanks to their computational and modeling strengths. Nonetheless, further research is needed to establish prediction error minimization as a core feature of brain function and to clarify its relationship to the properties of conscious experience (Mudrik et al., 2025).

## 8 Conclusion

Since the early experimental studies of the late 19th century, research on unconscious perception has been shaped by persistent methodological challenges and evolving experimental approaches aimed at demonstrating perception without awareness. In this review, we discussed some of the most relevant challenges researchers have faced in demonstrating unconscious perception, and examined how different measures of awareness (e.g., objective vs. subjective) may sometimes yield different awareness thresholds. To overcome these limitations, alternative approaches demonstrating unconscious perception have been developed. We further examined how emerging methodologies are shaping the study of unconscious perception. These approaches, while diverse in their implementation, share a common goal of disentangling perceptual sensitivity from conscious awareness, and we discuss their respective contributions as well as the conceptual and methodological limitations they entail. Finally, we emphasized the need for brain-based approaches to unconscious perception and discussed some promising studies in this area, while also highlighting the prominent role of individual differences and alternative frameworks such as predictive coding and active inference views in future research. Overall, the new approaches and methodologies discussed here will advance the field by addressing the challenges inherent in demonstrating cognition in the absence of awareness.

## Footnotes

^1^Note that in the present article, the terms ‘consciousness’ and ‘awareness’ will be treated interchangeably, referring to the subjective experience of perceiving a stimulus. Similarly, ‘unconscious perception’ and ‘unconscious processing’ will be also used as synonyms, the latter understood as unconscious stimulus processing.

^2^Jacoby’s Process Dissociation Procedure (PDP) (Jacoby, 1991) aims to separate and quantify conscious and unconscious influences on performance. The rationale behind the procedure is that unconscious processes are influenced by a briefly shown priming word. In an ‘inclusion task’, both unconscious and conscious processes contribute to reporting the target word. In contrast, in an ‘exclusion task’, unconscious processes lead to reporting the primed word, while conscious processes work to avoid it. By comparing performance across the two tasks, the relative contributions of conscious and unconscious processes can be assessed. The PDP has been criticized for being most useful for certain types of tasks (e.g., memory vs. perception; Visser and Merikle, 1999) and on the assumption that implicit and explicit processes operating independently (Curran and Hintzman, 1995). The exhaustiveness of the PDP has been also questioned, as knowledge revealed in the exclusion task (which is assumed to be unconscious) may be reported as being weakly conscious in subjective tests.

^3^Post-hoc data selection has been commonly used in unconscious perception studies when the overall group’s visibility is not at strict chance-level, making it impossible to infer a lack of awareness of the prime. In such cases, data from participants whose awareness scores fall below a specific threshold (usually chance-level in the prime visibility task) are analyzed separately. For this “unaware” group, their average performance score is then calculated. If significant priming effects are observed in this subset, unconscious perception is concluded. However, this method is controversial and strongly discouraged (Rothkirch et al., 2022; Shanks, 2017; though see Sklar et al., 2021 for an alternative perspective), as it can lead to regression to the mean (RttM). This statistical issue arises because selecting participants based on their awareness scores and then using those same scores to calculate mean awareness introduces a bias, essentially “double-dipping” and distorting the analysis due to non-random sampling of measurement errors (Rothkirch et al., 2022).

^4^Note that another source of variability in (un)consciousness research arises from the use of subjective awareness measures (Jimenez et al., 2025; Persuh, 2018), as discussed earlier, which rely on participants’ self-reports of conscious experience, typically on dichotomous or graded scales. These measures are influenced by two types of variability: a desired one, reflecting genuine differences in awareness thresholds across or within individuals, and an undesired one, stemming from variability in participants’ response criteria (Jimenez et al., 2025; Michel, 2023; Newell and Shanks, 2014). The latter undermines the validity of these measures, as it reflects decision biases rather than actual experience. This issue is particularly problematic when researchers select “unaware” trials *post hoc*, a common but flawed practice (Sabary et al., 2020; see Schmidt et al., 2024; Shanks, 2017 for critiques). To address this, Maniscalco and Lau (2012, 2014) proposed a method grounded in Signal Detection Theory that distinguishes between sensitivity and response bias in type 2 tasks—those involving confidence judgments. Their meta-*d’* metric quantifies how much information from the primary task reaches conscious awareness, enabling researchers to isolate genuine individual differences in conscious processing from mere variations in reporting criteria.

^5^Within this framework, attention plays a crucial—though distinct—role in enabling conscious access. Attention is conceptualized as the optimization of the expected precision of sensory signals, which fluctuates over time. It modulates the flow of information through the processing hierarchy, amplifying signals expected to be more reliable and down weighting those deemed less precise. As such, effort is allocated to sensory channels where low prediction errors are anticipated, resulting in a dynamic pattern shaped by the noise characteristics of incoming signals (Friston, 2009; Hohwy, 2013).
